# Identification and Regulation of Interleukin-17 (IL-17) Family Ligands in the Teleost Fish European Sea Bass

**DOI:** 10.3390/ijms21072439

**Published:** 2020-03-31

**Authors:** Carmen González-Fernández, Elena Chaves-Pozo, Alberto Cuesta

**Affiliations:** 1 Immunobiology for Aquaculture, Department of Cell Biology and Histology, Faculty of Biology, Regional Campus of International Excellence “Campus Mare Nostrum”, University of Murcia, 30100 Murcia, Spain; carmen.gonzalez1@um.es; 2 Oceanographic Centre of Murcia, Spanish Institute of Oceanography (IEO), Carretera de la Azohía s/n., Puerto de Mazarrón, 30860 Murcia, Spain; elena.chaves@ieo.es

**Keywords:** interleukin 17 (IL-17), Th17 cells, immunity, fish, nodavirus (NNV), PAMPs

## Abstract

Interleukin-17 (IL-17) cytokine comprises a family of six ligands in mammals with proinflammatory functions, having an important role in autoimmune disorders and against bacterial, viral, and fungal pathogens. While IL-17A and IL-17F ligands are mainly produced by Th cells (Th17 cells), the rest of the ligands are expressed by other immune and non-immune cells and have different functions. The identification of IL-17 ligands in fish has revealed the presence of six members, counterparts to mammalian ones, and a teleost-specific form, the fish IL-17N. However, tissue distribution, the regulation of gene expression, and scarce bioactivity assays point to similar functions compared to mammalian ones, though this yet to be investigated and confirmed. Thus, we have identified seven IL-17 ligands in the teleost European sea bass (*Dicentrarchus labrax*), for the first time, corresponding to IL-17A/F1, IL-17A/F2, IL-17A/F3, IL-17C1, IL-17C2, IL-17D, and IL-17N, according to the predicted protein sequences and phylogenetic analysis. They are constitutively and widely transcribed in sea bass tissues, with some of them being mainly expressed in the thymus, brain or intestine. Upon *in vitro* stimulation of head-kidney leucocytes, the mRNA levels of all sea bass IL-17 ligands were up-regulated by phytohemagglutinin treatment, a well-known T cell mitogen, suggesting a major expression in T lymphocytes. By contrast, the infection of sea bass juveniles with nodavirus (NNV), a very pathogenic virus for this fish species, resulted in the up-regulation of the transcription of IL-17C1 in the head-kidney and of IL-17C1 and IL-17D in the brain, the target tissue for NNV replication. By contrast, NNV infection led to a down-regulated transcription of IL-17A/F1, IL-17A/F2, IL-17C1, IL-17C2, and IL-17D in the head-kidney and of IL-17A/F1 and IL-17A/F3 in the brain. The data are discussed accordingly with the IL-17 ligand expression and the immune response under the different situations tested.

## 1. Introduction

The innate immune system uses several pattern-recognition receptors (PRRs) to recognize and interact with the common molecules present in pathogens, called pathogen-associated molecular patterns (PAMPs). PRRs signal the presence of infection and trigger the gene expression and synthesis of a broad range of molecules, mainly proinflammatory and chemotactic cytokines and antimicrobial peptides (AMPs) [1]. Cytokines are secreted proteins that act as key players and regulators of the immune response, making them crucial in the integration and coordination between innate and adaptive immune responses. In this scenario, the presence and function of T-helper (Th) lymphocytes (CD4^+^) is critical in mammals. The functions of Th cells range from the activation of the innate immune system, including B lymphocytes, cytotoxic T cells, as well as non-immune cells to the suppression of the immune reaction [2]. Upon activation, naïve Th cells differentiate into different subtypes. The main and classical types were Th1 cells, which produce interferon gamma (IFNγ), interleukin (IL)-2, and tumor necrosis factor-α (TNFα), and are important for antiviral and antibacterial cellular immunity, whereas Th2 cells produce IL-4, IL-5, and IL-13, which trigger the humoral immune response and are important against extracellular pathogens [3]. Although other Th populations have been described during the last two decades in mammals, including follicular helper T cells (Tfh), induced T-regulatory cells (iTreg), the regulatory type 1 cells (Tr1), or the potentially distinct T-helper 9 (Th9), the Th17 cell subpopulation received particular attention. Th17 cells are activated by IL-23 and secrete IL-17A, IL-17F, IL-21, IL-22, and granulocyte-macrophage colony-stimulating factor (GM-CSF) cytokines [4]. Mammalian IL-17 is a cytokine family that comprises six ligands, from A to F. IL-17A and IL-17F ligands, mainly produced by Th17 cells and used as their hallmark, are considered pivotal proinflammatory cytokines in autoimmune disorders and in the immunological response towards pathogens, playing a special role in the regulation of the gut microbiota and hence in the first line of defense in the mucosal tissues [3]. However, other lymphocyte subtypes, such as CD8^+^ T cytotoxic, natural killer T (NKT), γδT, or innate lymphoid cells, also produce IL-17A and F cytokines [5]. The other four members of the IL-17 family are not related to the Th17 cells, and their role is still uncertain in mammals [5]. IL-17E, called IL-25, is produced by innate immune and respiratory tract epithelial cells and plays an important function in allergy. IL-17C is expressed by epithelial cells, CD4^+^ T cells, dendritic cells, and macrophages upon inflammatory insults. IL-17B is widely produced in many cell types, including chondrocytes, neurons, intestinal epithelial cells, and breast cancer cells, whilst IL-17D is detected in various tissues, including brain, heart, lung, pancreas, skeletal muscle, and adipose tissue, but, in the immune system, IL-17D is restricted to naïve CD4^+^ T and B cells [5]. However, their functions are not sufficiently clarified in mammals. In fact, actual research is focusing on the functions and regulation of IL-17 cytokines in pathological situations, including infectious diseases and autoimmune disorders.

Fish are the first group in the animal evolution that possess both innate and adaptive immunity. Fish show Th cells (CD4^+^), and functional and genomic studies greatly suggest that fish possess very similar populations of Th cells compared to humans, though they might be less evolved [6]. Regarding the Interleukin-17 (IL-17) cytokine family, up to seven IL-17 ligands have been identified in several fish species, including rainbow trout (*Oncorhynchus mykiss*) [7,8], Atlantic salmon (*Salmo salar*) [9,10], zebrafish (*Danio rerio*) [11], fugu (*Takifugu rubripes*) [12], medaka (*Oryzias latipes*) [13], channel catfish (*Ictalurus punctatus*) [14], miiuy croaker (*Miichthys miiuy*) [15], common carp (*Cyprinus carpio*) [16,17], yellow croaker (*Larimichthys crocacea*) [18], grass carp (*Ctenopharyngodon idella*), [19,20] and Siberia sturgeon (*Acipenser baerii*) [21]. Overall, transcription studies detect a wide and constitutive expression of IL-17 ligands in several tissues but show a tissue–ligand–specific pattern. Interestingly, the expression of these cytokines is especially abundant in mucosal and immune tissues, pointing to an important role in immunity in the first entry sites (skin, gills and intestine) for pathogens. In addition, their expression is regulated by lipopolysaccharide (LPS) administration, as well as upon bacterial and viral challenges, pointing to their role in the inflammatory response. Interestingly, fish IL-17A and F ligands are orthologues to mammalian counterparts and show the same genomic organization and are therefore named as IL-17A/F, which comprises up to three ligands [11,13]. Strikingly, fish IL-17D shows the greatest conservation and is proposed as the most ancient IL-17 appearing in the vertebrate evolution [15]. While IL-17E is only present in mammals [15], a fish-specific IL-17 ligand, called IL-17N, has been described in genomic and transcriptomic studies [13]. This fish IL-17N is well conserved in the key residues of the IL-17 family and is only present in bony fish, being undetected in jawless and chondrichthyan fish [13,15]. Based on the very scarce bioactivity studies performed in fish, IL-17 ligands seem to have an important role in the inflammatory response in a similar way to mammalian IL-17A and F. Thus, recombinant IL-17A/F2 increase the transcription of IL-8, IL-6, and AMP genes in rainbow trout [7] or common carp [16] leucocytes. Similarly, croaker recombinant IL-17C was an active inducer of chemokine, proinflammatory cytokine, IFNγ, and AMP genes’ expression via the NF-kB promoter, whilst recombinant IL-17D did it to a much lower extent [18]. Similar functions were also observed for grass carp (*Ctenopharyngodon idella*) IL-17A/F1 and IL-17D recombinant proteins [19,20]. Further studies are needed to ascertain the functional role of fish IL-17 ligands and their implication in immunity.

Understanding the fish IL-17 biology would be beneficial from both evolutionary and practical perspectives. Thus, the objective of this work consisted of the identification and transcriptional evaluation of IL-17 ligands in the European sea bass (*Dicentrarchus labrax*), a bony fish species of great economic importance for the Mediterranean aquaculture industry. This species is very sensitive to numerous pathogenic viruses, bacteria, fungi, and parasites [22], causing serious infectious diseases and economic losses. Among all the pathologies, the most recognized pathogen for sea bass is the nervous necrosis virus (NNV), which causes viral encephalopathy and retinopathy, reaching the 100% of mortality in juvenile and larvae stages [23,24]. NNV replicates in the central nervous system (brain, spinal cord, and eye) and causes abnormal behaviour, related to erratic swimming [25], and finally death. Previous studies have evidenced a strong expression of proinflammatory cytokines and AMP genes in the brain of European sea bass in response to NNV infection [25,26,27]. Taking all this into consideration, we have identified seven IL-17 ligands of European sea bass in silico and evaluated their transcription in tissues from naïve specimens, their regulation in head-kidney (the main hematopoietic tissue in fish and equivalent to the mammalian bone marrow) leucocytes upon mitogen, PAMPs and pathogen exposure *in vitro*, as well as in the brain and head-kidney upon NNV infection *in vivo*. The results are discussed trying to ascertain their role in fish immunity and regulation during infection.

## 2. Results and Discussion

### 2.1. Identification of IL-17 Ligands

**Figure 1:**
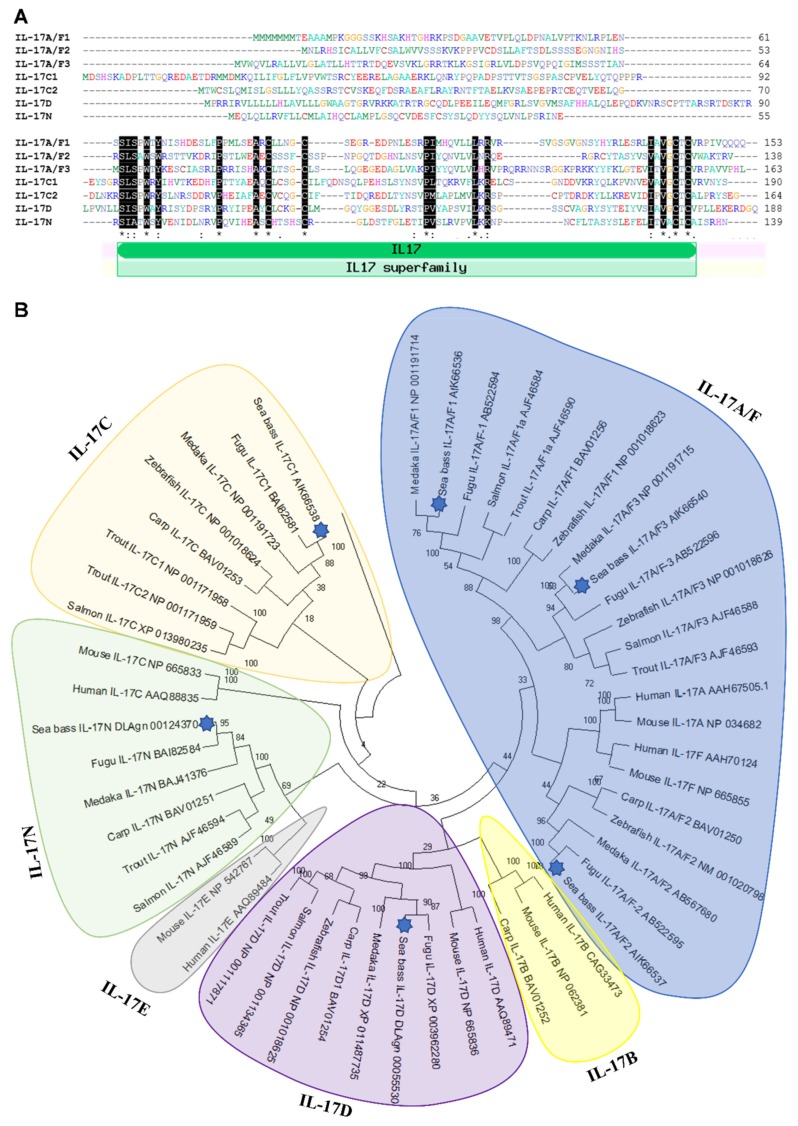
Identification of seven IL-17 ligands in European sea bass. (**A**) The predicted amino acid sequences of European sea bass IL-17 were aligned by ClustalW. The dashes denote gaps introduced to maximize alignments. Identical and similar residues are indicated by asterisks and colons, respectively. The position of the IL-17 domain is indicated. (**B)** The neighbour-joining tree of the European sea bass IL-17 deduced proteins with fish and mammalian orthologues. The genetic distances were calculated based on protein differences (p-distance) with complete deletion of gaps. The number at each node indicates the percentage of bootstrapping after 1000 replications. Accession numbers are indicated.

Seven IL-17 cytokine members (IL-17A/F1, IL-17A/F2, IL-17A/F3, IL-17C1, IL-17C2, IL-17D, and IL-17N) have been identified in European sea bass at the gene level. We failed to find IL-17B and IL-17E orthologues in European sea bass genomic and transcriptomic resources, which is supported by the lack of IL-17E in all fish species studied so far and the scarce and controversial identification of IL-17B, only found in channel catfish and cave fish [12,15]. Protein sequences were predicted from the mRNA sequences and were annealed to identify the conservation of key residues (Figure 1A). 

Although some of the identified sequences are partial, all of them show a variable sequence at the N-terminus followed by a very well conserved IL-17 domain (Figure 1A), for which sea bass sequences are classified as IL-17 members in bona fide. This domain contains four conserved cysteines, to form two disulphide bonds, as the rest of vertebrate IL-17 members [28,29]. In addition, all of them, except sea bass IL-17A/F1, also show a fifth cysteine residue. Interestingly, according to the Signal P analysis, sea bass IL-17A/F1 and IL-17C1 reveal a lack of signal peptide, suggesting that they are not released cytokines, as also found for other putative IL-17 members in frogs and invertebrates [29]. 

The relationship of sea bass IL-17 proteins with other members of the IL-17 family was investigated with the construction of a phylogenetic tree. The analysis revealed that sequences of sea bass IL-17 ligands are tightly clustered with teleost fish, mainly with fugu and medaka orthologues (Figure 1B). Our data suggest that the sea bass IL-17A/F and IL-17C forms are related and that IL-17D and IL-17N are too, which is also confirmed in previous studies [12,15] but not in others [13,14,29]. As also documented, fish IL-17A/F1 and IL-17A/F3 are more related to mammalian IL-17A and IL-17F than fish IL-17A/F2 [13]. A fact that seems clear is that IL-17D appeared in fish and diverged during evolution since this is the most well-conserved [15]. Further and deeper analysis of fish IL-17 members would help to clarify the phylogeny and evolution of this cytokine family. 

### 2.2. Tissue Distribution of IL-17 mRNA 

**Figure 2:**
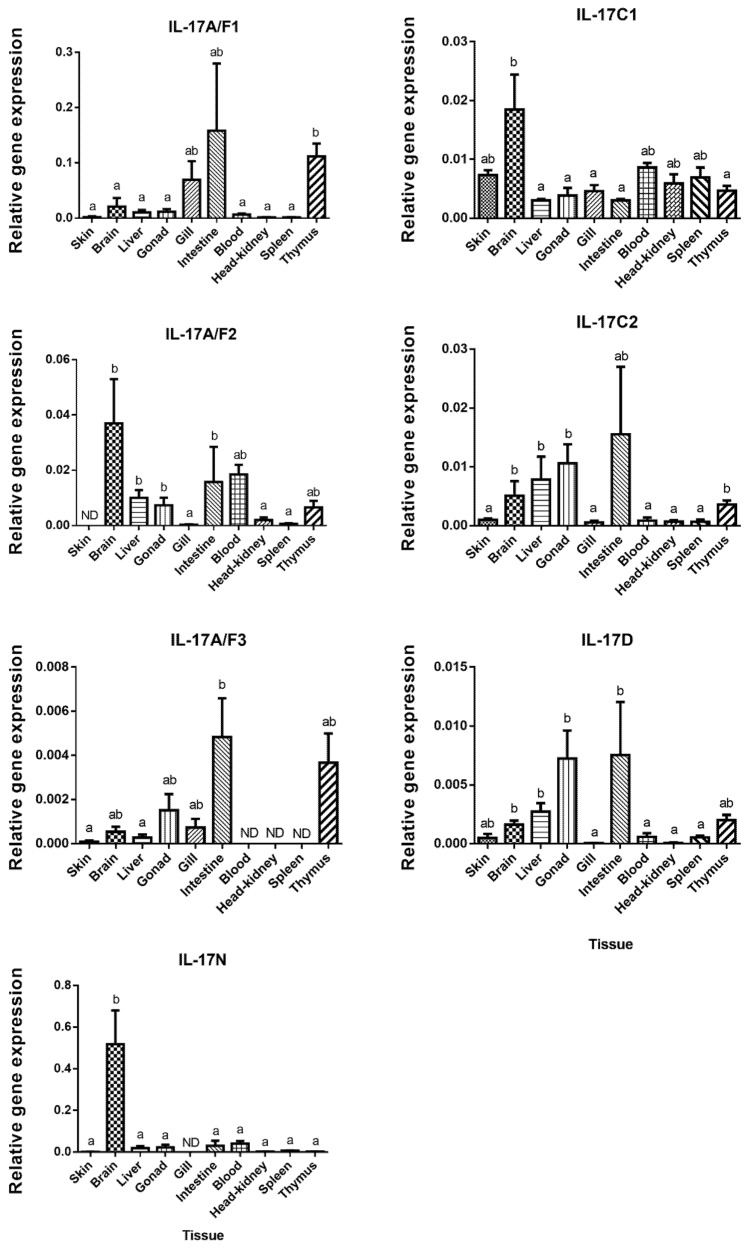
European sea bass Interleukin-17 (IL-17) transcripts are widely and constitutively expressed in the tissues. The relative gene expression of IL-17 ligands in the tissues of naïve European sea bass specimens. The data are presented as the mean (*n*= 3) ± SEM relative to the expression of the endogenous controls. Different letters indicate differences in expression between tissues (ANOVA, *p* < 0.05). ND, undetected.

The tissue distribution of the IL-17 family ligands in European sea bass has been related with their biological role, although the function of these cytokines has not yet been completely elucidated and understood in fish [29]. In our study, IL-17N and IL-17A/F1 mRNA are the highest expressed ligands in the brain and in the intestine, respectively (Figure 2). 

European sea bass IL-17A/F1 and IL-17A/F3 transcripts show a close pattern of tissue distribution with the highest expression in the intestine and thymus. These two forms are related to the mammalian IL-17A and F forms, which are mainly produced by Th17 cells [4] and justifies their great expression in the sea bass thymus. By contrast, IL-17A/F3 was not identified in the sea bass blood, head-kidney (HK), or spleen, which are also important lymphoid tissues in fish [30], whereas IL-17A/F2 shows high expression in the brain, blood, liver, and gonad (Figure 2). Unfortunately, great differences in the tissue distribution in the fish species studied so far have been documented, which makes difficult to ascribe potential roles for fish IL-17 ligands. For example, the highest expression of the IL-17A/F1 and IL-17A/F2 genes has been documented in the intestine of zebrafish, in the brain of medaka and fugu, or in the spleen of common carp [13,17]. Interestingly, the transcription of the rainbow trout IL-17A/F2 gene was very high in the intestine and gills, suggesting a potential role in the mucosal immunity [7]. Pointing to this suggestion, we have also found high transcript levels of IL-17A/F forms in the sea bass intestine, and of 17A/F1 and 17A/F3 in the gills. However, our data about the skin transcription does not support this hypothesis. Regarding the IL-17C1 mRNA expression, this ligand is observed in all sea bass tissues, but it shows a significant high level in brain, followed by skin, blood, HK, and spleen (Figure 2), as also occurs in rainbow trout [8]. In sea bass, the expression of the IL-17C2 gene differs from the IL-17C1 gene expression, as IL-17C2 presents high expression levels in the thymus, liver, brain, intestine, and gonad (Figure 2). For comparison with other species, the highest gene expression level of IL17C forms is observed in the gills for zebrafish IL-17C, miiuy croaker IL-17C, and fugu IL-17C2, as well as in the head-kidney for fugu IL-17C1, in the gonad for channel catfish IL-17C, and in the blood and intestine for miiuy croaker IL-17C [12,13,14,15]. European sea bass IL-17D gene is expressed in all the studied tissues, with high levels in the intestine, gonad, liver, brain, and thymus (Figure 2), in a similar way to zebrafish, fugu, and Atlantic salmon [10,13]. However, in channel catfish, the IL-17D transcripts are only detected in the ovary [14], while in miiuy croaker, this cytokine is lacking in the liver, stomach, eye, or spleen [15]. Finally, tissue distribution pattern of IL-17N in European sea bass is totally different to the other IL-17 ligands, being mostly expressed in the brain (Figure 2). This IL-17N gene expression shows important differences among fish species but it is usually highly expressed in the brain and HK [12,13,29]. Overall, important differences in the tissue distribution of fish IL-17 ligands suggest tissue- and species-specific roles that needs to be elucidated.

### 2.3. IL-17 mRNA Is Up-Regulated In Vitro by the T Cell Mitogen PHA

**Figure 3:**
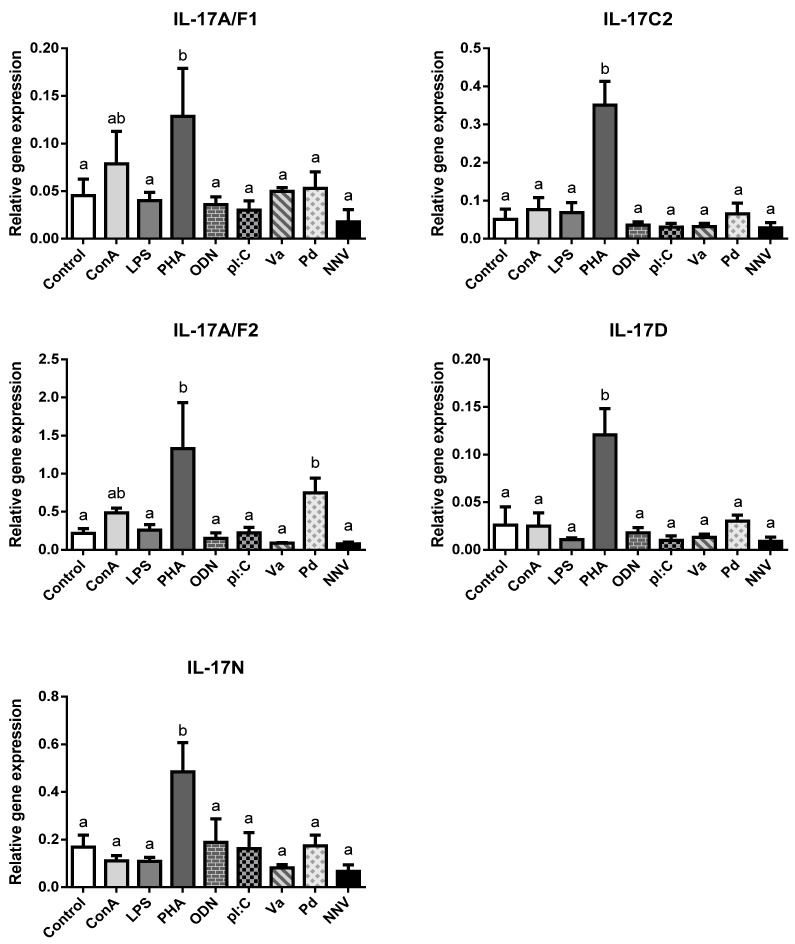
**Transcription of European sea bass IL-17 ligands is induced in head-kidney leucocytes by T cell mitogens**. The relative gene expression of IL-17 ligands in the European sea bass head-kidney leucocytes incubated for 4 h with culture medium (Control), 5 μg/mL concanavalin A (ConA), 5 μg/mL lipopolysaccharide (LPS), 10 μg/mL phytohemagglutinin (PHA), 50 μg/mL synthetic unmethylated cytosine-phosphodiester-guanosine oligodeoxynucleotide 1668 (ODN), 25 μg/mL PolyI:C (pI:C), 10^8^ heat-killed *Vibrio anguillarum* (Va) or *Photobacterium damselae* (Pd) bacteria/mL, and 10^6^ TCID_50_ nodavirus (NNV)/mL. The data are presented as the mean (*n* = 5) ± SEM relative to the expression of the endogenous controls. Different letters indicate differences in gene expression (ANOVA, *p* < 0.05).

IL-17 cytokines are key regulators of the inflammatory response in mammalian immunity though this has not been confirmed in fish so far. In order to ascertain the potential role of IL-17 ligands in the immune responses of European sea bass, leucocytes isolated from the head-kidney were exposed for 4 h to several mitogens, PAMPs, or pathogens and the IL-17 gene transcription levels evaluated (Figure 3). 

We failed to detect IL-17A/F3 gene expression in any treated HK leucocytes, as also occurs in the HK from naïve specimens (Figure 2). In addition, neither of the IL-17C1 expression levels were detected. Notwithstanding this, our results show an important and statistically significant up-regulation in the transcription of all sea bass IL-17 ligands upon stimulation with PHA, and scarce but non-significant regulation of IL-17A/F1 and IL-17A/F2 genes with ConA (Figure 3). Both PHA and ConA are well-known stimulators of T-lymphocytes [31], so our data suggest that sea bass T cells are being activated to transcript IL-17 ligands. Although the precise cell-types expressing fish IL-17 cytokines are unknown, it seems that other HKLs different from T cells might not express them since other stimuli failed to regulate their transcription. Interestingly, considering that Th lymphocytes are important producers of IL-17 cytokines, only two studies have evaluated the IL-17 gene regulation of fish leucocytes by T cell mitogens or activators. Thus, rainbow trout HKLs showed increased transcription of IL-17A/F2 upon PHA stimulation [7], while Atlantic salmon HKLs up-regulated IL-17A/F1 and IL-17A/F2 [9]. By contrast, most of the studies that evaluated the transcription of fish IL-17 ligands *in vitro* used LPS, which is considered a mitogen for B-lymphocytes [32]. Thus, although we failed to detect any alteration in the sea bass transcription of IL-17 ligands upon the LPS stimulation of HKLs (Figure 3), rainbow trout IL-17A/F2, IL-17C1, and IL-17C2 and all fugu IL-17s did [7,8,12]. Interestingly, LPS injection up-regulated the IL-17 gene transcription in zebrafish [11] but failed to do so in the miiuy croaker [15]. In addition, pI:C, a well-known agonist of RNA viral infections, failed to alter the IL-17 gene expression in the sea bass HKLs (Figure 3) but up-regulated the IL-17C1 and IL-17C2 expression in rainbow trout [8] and IL-17A/F3 in Atlantic salmon *in vitro* [9], as also occurred with the IL-17A/F1, IL-17A/F2 and IL-17N gene expression in miiuy croaker upon injection [15]. On the other hand, Siberia sturgeon IL-17D gene expression was down-regulated by pI:C [21]. In addition, rainbow trout and Atlantic salmon IL-17 gene expression was up-regulated upon *in vitro* stimulation with phorbol 12-myristate 13-acetate (PMA), calcium ionophore, or fish cytokines [7,8,9]. Further *in vitro* studies would help to know and understand the IL-17 biology in fish.

We have also evaluated the transcription of sea bass IL-17 ligands after HKL incubation with pathogenic bacteria and virus. They show little regulation except an increase in the IL-17A/F2 mRNA levels upon incubation with killed *P. damselae* bacteria (Figure 3). Although there is no information at this respect in fish in vitro, the in vivo injection with different bacteria or virus resulted in the regulation of the transcription of several IL-17 ligands in catfish [14], rainbow trout [7,8], Siberia sturgeon [21], common carp [17], yellow croaker [18], and Atlantic salmon [9]. In mammals, IL-17 ligands play an important role against both intracellular and extracellular bacterial infections, acting as proinflammatory mediators that can also synergize with other cytokines and interferons to increase the induction of proinflammatory responses from various target cells [33]. Thus, IL-17A and IL-17F are essential for the control of extracellular and intracellular bacterial infections in the early stages (<7 days), while in the later stages IL-17F becomes more important [34]. Although further studies are needed to evaluate this hypothesis in fish, recombinant fish IL-17A/F1, IL-17A/F2, IL-17C, or IL-17D forms are able to induce the expression of inflammatory cytokines and AMPs, probably through the NF-kB pathway [7,16,18,19]. 

### 2.4. IL-17 Genes Are Differently Regulated by NNV Infection

**Figure 4:**
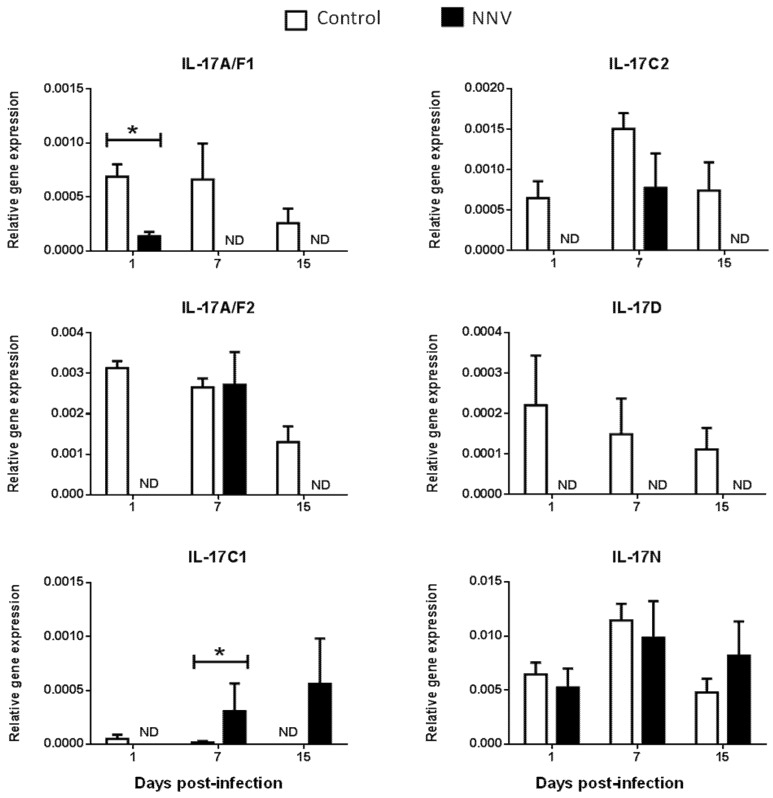
**Nodavirus regulates the transcription of European sea bass IL-17 genes in the head-kidney**. The relative gene expression of IL-17 ligands in the head-kidney of European sea bass specimens intramuscularly injected with 100 µL of culture medium alone (control) or containing 10^6^ TCID_50_ of nodavirus (NNV)/fish. The data are presented as the mean (*n*= 4–6) ± SEM relative to the expression of the endogenous controls. Asterisks denote significant differences with the control group at each sampling time (*Student-t*-test, *p* < 0.05). ND, undetected.

**Figure 5:**
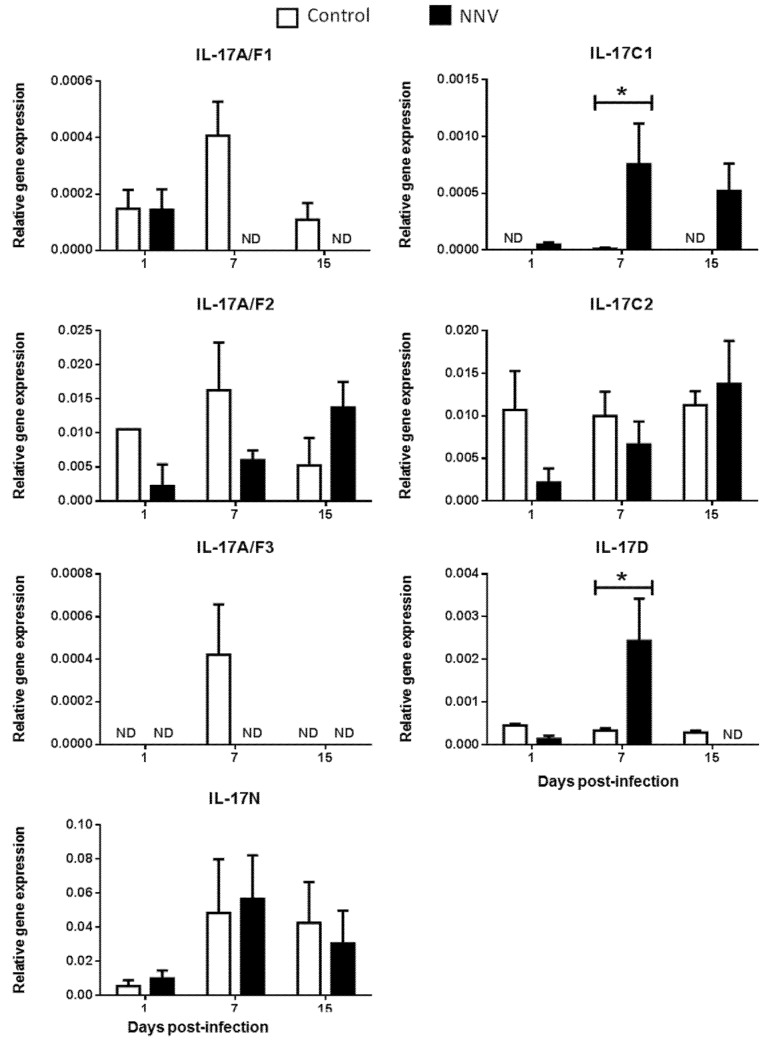
Nodavirus regulates the transcription of European sea bass IL-17 genes in the brain. The relative gene expression of IL-17 ligands in the brain of European sea bass specimens intramuscularly injected with 100 µL of culture medium alone (control) or containing 10^6^ TCID_50_ of nodavirus (NNV)/fish. The data are presented as the mean (*n*= 4–6) ± SEM relative to the expression of the endogenous controls. Asterisks denote significant differences with the control group at each sampling time (*Student-t*-test, *p* < 0.05). ND, undetected.

It is well known that, during infection, pathogens rapidly trigger inflammatory responses through several mechanisms, including the activation of proinflammatory cytokines [35]. Viruses may simultaneously activate multiple IL-17-producing cell subsets that differ in several key biological activities [33]. Strikingly, in mammals, IL-17 ligands have a dual positive and negative protective role against viral infections [36]. However, the inflammatory processes triggered by viral infections in fish are less known. To investigate the regulation of these genes upon viral exposure, the gene expression levels of IL-17 ligands were analysed in European sea bass infected with a lethal dose of NNV [37] in the main hematopoietic tissue, the HK (Figure 4), and in the target tissue for NNV replication, the brain (Figure 5). NNV-infected fish rapidly started to show signs of disease, such as erratic swimming, and the first death was registered after 2 days of infection, reaching an accumulated fish death of 55% after 17 days of infection [37].

We did not determine the baseline expression levels before the NNV infection in the same fish specimens, and, therefore, we cannot know whether these gene expression levels are modified by the management and injection process. Thus, we compared the transcription levels in fish injected with NNV with those injected with phosphate buffer saline (PBS) alone at the same sampling time. In the HK, as previously seen in Figure 2, there is no expression of the IL-17A/F3 gene in control nor in NNV-infected specimens. Although the expression levels are very low, there is an important down-regulation in the transcription of IL-17A/F1, IL-17A/F2, IL-17C2, and IL-17D genes in the sea bass HK upon infection with NNV (Figure 4), which decrease from low to undetectable levels. By contrast, the transcription of the IL-17C1 gene is up-regulated at 7 and 15 days post-infection in the HK. In the brain, down-regulations in the expression of sea bass IL-17A/F1 and IL-17A/F3 genes are observed upon NNV infection while IL-17C1 and IL-17D mRNA levels are significantly up-regulated (Figure 5). Interestingly, the expression trend of sea bass IL-17A/F1, IL-17C1, IL-17D, and IL-17N genes in the HK and brain is very similar upon NNV infection. It is noteworthy that a reduction or a total blockage of the expression of the IL-17A/F1 and IL-17D transcription was registered in both tissues. However, the IL-17C1 transcription is up-regulated, suggesting that this gene is strongly associated with NNV infection. Our results demonstrate the complexity of IL-17 biology in fish and could be explained by the presence of the certain control of the inflammation in the brain as well as by the mobilization of leucocytes from the HK to the brain during the infection. To ascertain this, further investigations are necessary. During viral infection, the high production of IL-17A, IL-17F, and IL-22 has been directly related with inflamed tissues [38]. The role of IL-17 in mammals has been related to proinflammatory properties that make IL-17 an essential mediator of inflammation and immunopathology [36]. This would imply a proinflammatory state due to viral infection. However, our data about IL-17 suggest a low degree of inflammation in sea bass tissues after NNV infection. In fact, this is corroborated by previous studies showing that NNV produces very little and transitory inflammation in the sea bass, as determined by the increment in the transcription of IL-1β, TNFα, or IL-6 cytokines [26,39]. In sharp contrast, IL-17 has also been reported to participate in suppressing local or systemic inflammation during viral infections [36], which could potentially help to maintain tissue integrity [40]. It is essential for survival to maintain a balance between pathogenic and protective functions of IL-17 [40]. Related to this, our data suggest that the constitutive expression of sea bass IL-17N, IL-17A/F2, and IL-17C1 genes in the brain (Figure 5) might be related to the suppression of a strong inflammatory response. Moreover, IL-17A/F2 and IL-17N cytokine gene expression kept steady upon NNV infection, while the expression of IL-17C1 increased at 7 days post-infection. Although some up- and down-regulations in the expression of fish IL-17 family members are triggered by the RNA viral analogue pI:C [8,9,15,21], very few data exist regarding a viral infection. Thus, viral infection increased the transcription of IL-17A/F2 in rainbow trout [40], while members of the IL-17 pathway have been enriched upon reovirus infection [41]. However, this is not clear because virus-infected fish produce IFN, and the incubation of fish leucocytes with recombinant IFNγ resulted in unaltered or decreased IL-17 gene expression [9]. The understanding of these mechanisms could help to improve strategies against pathogenic infections in fish, which are of great interest in aquaculture.

## 3. Materials and Methods 

### 3.1. Animal Maintenance and Sampling

European sea bass (*Dicentrarchus labrax L.)* specimens were bred at the Spanish Institute of Oceanography (IEO, Mazarrón) and transported to the aquaria at the University of Murcia. The fish were maintained in closed flow-through marine aquaria (28% salinity, 24 ± 2 °C, 12 h light: 12 h dark photoperiod) with suitable aeration and filtration systems and were fed daily with a commercial diet (Skretting, Burgos, Spain). Juvenile specimens with a mean body weight (bw) of 305 ± 77 g were used for the in vivo experiment, while specimens of 325 ± 37 g were used for the expression in naïve tissues and the in vitro study. The fish were anesthetized with 40 μL/L of clove oil before sampling, then weighed, completely bled, and immediately decapitated. All the experiments fulfilled the Guidelines of the European Union Council (2010/63/UE) and protocols were approved by the Committee on the Ethics of Animal Experiments of the IEO (Permit Number: 2010/02) and of the University of Murcia (Permit Number: A13150104).

In order to analyse the constitutive gene expression in naïve conditions, brain, gill, liver, skin, gonad, gut, head-kidney (HK), spleen, thymus, and blood from 3 healthy fish were sampled and immediately frozen in TRIzol^®^ Reagent (Life Technologies, California, USA) and kept at −80 °C.

### 3.2. In Vitro Treatments

European sea bass head-kidney leucocytes (HKLs) (*n* = 5 fish) were obtained and processed independently as previously described [42] and maintained in Leibovitz’s L-15 medium (Gibco, California, USA) supplemented with 10% foetal bovine serum (FBS), 2 mM glutamine, 100 IU/mL penicillin, 100 µg/mL streptomycin, and 20 mM HEPES (Gibco). Later, HKLs were exposed through incubation of 10^7^ HKLs/mL in 48-well microtiter plates (Nunc, New York, USA) at 22 °C during 24 h with: culture L-15 medium (control treatment), 5 μg/mL concanavalin A (ConA; Sigma-Aldrich, Darmstadt, Germany), 5 μg/mL lipopolysaccharide (LPS; Sigma-Aldrich), 10 μg/mL phytohemagglutinin (PHA; Sigma-Aldrich), 50 μg/mL synthetic unmethylated cytosine-phosphodiester-guanosine oligodeoxynucleotide 1668 (CpG ODN; sequence 5′-TCCATGACGTTCCTGATGCT-3′; Eurogentec, Seraing, Belgium), 25 μg/mL Poly I:C (pI:C; Sigma-Aldrich), 10^8^/mL of *Vibrio anguillarum* (Va), or *Photobacterium damselae* (Pd) heat-killed bacteria and 10^6^ TCID_50_ NNV/mL. After exposure, HKLs were washed with phosphate buffer saline (PBS) and conserved in TRIzol^®^ Reagent at -80°C.

### 3.3. In Vivo Infection with NNV

Juvenile specimens of sea bass were randomly distributed in two tanks of 500 L (28% salinity, 24 ± 2 °C, 12 h light: 12 h dark photoperiod) and acclimatized for 15 days prior to the infection. Infection was carried out by intramuscular injection with 100 µL containing 10^6^ TCID_50_ of NNV/fish (strain 411/96, genotype RGNNV), while the other group of fish served as a control and was injected with 100 µL of culture medium [37]. The fish were sampled at 1, 7, and 15 days post-infection and the brain and HK of each fish (*n* = 4–6 fish) was extracted and immediately frozen in TRIzol^®^ Reagent and kept at −80 °C.

### 3.4. Real-Time PCR Analysis

Total RNA was isolated from TRIzol^®^ Reagent frozen samples following the manufacturer’s instructions. Total RNA was treated with DNAse I (Promega, Wisconsin, USA) to remove genomic DNA and the first strand of cDNA synthesized by reverse transcription using the Superscript III reverse transcriptase (Life Technologies, California, USA) according to the manufacturer’s instruction. Real-time PCR was performed using a 7500 Fast Real Time PCR System (Roche Applied Science, Indianapolis, USA) and SYBR Green PCR Core Reagents (Applied Biosystems, Wisconsin, USA). The reaction mixtures were incubated at 95 °C for 10 min, followed by 40 cycles of 15 s at 95 °C, 1 min at 60 °C, and finally 15 s at 95 °C, 1 min at 60 °C, and 15 s at 95 °C. The gene expression was corrected by the geometric mean of the elongation factor 1 alpha (Ef1α) and ribosomal 18S expression as house-keeping genes. Relative mRNA quantities of the target in each sample was normalized to the expression of the reference genes [43]. The primers are listed in Supplementary Table S1. Negative controls with no sample were always included in the reactions.

### 3.5. Bioinformatic Analysis

Sequences for IL-17 ligands were searched in internal RNA-seq experiments [44,45] and in the European sea bass genome project database (http://seabass.mpipz.mpg.de/). The identified mRNA sequences were detected, and putative proteins were predicted within the ExPASy Molecular Biology server (http://us.expasy.org). Protein sequences of IL-17 ligands were analysed with the BLAST algorithm (http://blast.ncbi.nlm.nih.gov/Blast.cgi) to search homologous sequences, and a phylogenetic tree was constructed by MEGA 7.0 program with the bootstrap value set to 1,000 replicates [46]. 

### 3.6. Statistical Analysis

Statistical analysis was performed using STATGRAPHICS centurion XVII (Statpoint Technologies, Virginia, USA). Differences were considered significant at *p* < 0.05. One-way-ANOVA analysis was performed to establish significant differences in gene expression in the naïve tissues and the in vitro treatments of HKLs. The normality and homogeneity of variances of the data distribution were tested by standardized skewness and standardized kurtosis and Levene’s test, respectively. Tukey’s post hoc test was used to test differences if required. Additionally, Student-t-test was used to identify significant differences in gene expression in the brain and HK at each sampling time upon exposure to NNV in vivo.

## 4. Conclusions

This is the first study identifying, at gene level, the IL-17 ligands in the European sea bass teleost fish and evaluating their regulation upon *in vitro* and *in vivo* insults. Our results reveal the presence of seven IL-17 ligands with a very-well-conserved IL-17 domain. Most of the ligands show constitutive expression and a wide distribution in European sea bass naïve tissues with high transcription levels in the thymus, intestine, and brain, but sharp tissue- and ligand-specific profiles are detected. *In vitro*, sea bass HKLs are induced to up-regulate the transcription of IL-17 ligands by PHA, suggesting that these cytokines are mainly produced by T cells. Although we do not know how the basal expression levels are modified by the injection process, the infection with the pathogenic NNV up-regulates the IL-17C1 transcription in both HK and brain, and the IL-17D in the brain. In sharp contrast, IL-17A/F1, IL-17A/F2, IL-17C2, and IL-17D transcription was inhibited in the HK, while IL-17A/F1 and IL-17A/F3 did in the brain upon NNV infection. These findings point to a tight control of the inflammatory response in the brain, as well as the leucocyte mobilization to the brain, in NNV-infected fish. Further studies dealing with the source and regulation of IL-17 could provide some light for understanding how the fish immune system modulates the process of inflammation upon infection, which could help to improve the strategies to prevent or to fight pathogens. 

## Supplementary Materials

Supplementary materials can be found at https://www.mdpi.com/1422-0067/21/7/2439/s1.
