# Supplementary material for: Identification and Regulation of Interleukin-17 (IL-17) Family Ligands in the Teleost Fish European Sea Bass
Source: Int J Mol Sci. 2020 Mar 31;21(7):2439. doi: 10.3390/ijms21072439 (PMC7178287; doi:10.3390/ijms21072439)
Supplement: Supplementary file 1 [file ijms-21-02439-s001.pdf]

Supplementary Table 1. Primers used for analysis of gene expression by real-time PCR in this study.

Name	Gene	Fw 5'-3'	Rev 5'-3'	Accesion number
Interleukin-17A/F1	IL-17A/F1	GACTCAGAGGGCAGAGAGGA	CGGTAGTGGTAGCTGTTCCC	AIK66536
Interleukin-17A/F2	IL-17A/F2	AACTCGGTCCCCATCTACCA	TTTGGCCCAGACACAGGTAC	AIK66537
Interleukin-17A/F3	IL-17A/F3	CCAGGTCCTAGTCCTCCACA	TATCACCTCCGTCCCCAGTT	AIK66540
Interleukin-17C1	IL-17C1	CTGTGTTCCGGATGCATCCT	CTGCAGAGCTCCCTCTTGAG	AIK66538
Interleukin-17C2	IL-17C2	GTCTGTCAGGGCTGCATCAT	TATTTATCGCGGTCGGTCGG	AIK66539
Interleukin-17D	IL-17D	CGGTATCCACGCTACATCCC	AGGATCACAGAGGGAGCGTA	DLAgn_00055530
Interleukin-17N	IL-17N	GAGTGCCTCAGGTCATCCAC	TTTCTTGAGGACAGGCACCC	DLAgn_00124370
Elongation factor 1 alpha	Ef1α	TCCCTGGAGAAGAGCTACGA	AGGAAGGAAGGCTGGAAAAG	AJ537421
Ribosomal protein S18	18S	TTCCTTTGATCGCTCTTAACG	TCTGATAAATGCACGCATCC	AY831388
